# Biological inflammatory markers mediate the effect of preoperative pain-related behaviours on postoperative analgesics requirements

**DOI:** 10.1186/s12871-015-0167-9

**Published:** 2015-12-16

**Authors:** Myriam Daoudia, Céline Decruynaere, Bernard Le Polain de Waroux, Jean-Louis Thonnard, Léon Plaghki, Patrice Forget

**Affiliations:** Department of Anesthesiology, Cliniques universitaires Saint-Luc, Université catholique de Louvain, Brussels, Belgium; Haute Ecole Leonard de Vinci, Institut Parnasse-Deux Alice, Brussels, Belgium; Institute of Neuroscience (pole COSY), Université catholique de Louvain, Brussels, Belgium

## Abstract

**Background:**

The predictive value of an individual’s attitude towards painful situations and the status of his immune system for postoperative analgesic requirements are not well understood. These may help the clinician to anticipate individual patient’s needs.

**Methods:**

Sixty patients, who underwent a laparoscopic cholecystectomy under standardised general anaesthesia, were included. The total analgesic requirements during the first 48 h were the primary endpoint (unitary dosage, UD). The individual’s attitude towards imaginary painful situations was measured with the Situational Pain Scale (SPS). The emotional status was assessed by the Hospital Anxiety and Depression Scale (HADS) and the inflammatory status by the neutrophil-to-lymphocyte ratio (NLR).

**Results:**

Univariate analyses revealed a significant association between UD and SPS, HADS and NLR. A negative relationship between SPS and NLR (NLR = 0.820–0.180*SPS;R^2^ = 0.211;*P* < 0.001) and a positive relationship between SPS and HADS (HADS = 14.8 + 1.63*SPS; R^2^ = 0.159;*P* = 0.002) were observed. A multiple linear regression analysis showed that the contribution of NLR to the UD was the most effective. A mediation analysis showed a complete mediation of the effect of SPS on UD (R^2^ = 0.103;*P* = 0.012), by the NLR (SPS on NLR: R^2^ = 0.211;*P* = <0.001), the HADS (SPS on HADS: R^2^ = 0.159;*P* = 0.002). The variance in UD explained by the SPS was indirect and amounts to 46 % through NLR and to 34 % through HADS.

**Conclusions:**

In this series, preoperative pain-related attitudes (SPS) were associated with the postoperative analgesic requirements (UD) after a cholecystectomy. Eighty per cent of this effect was mediated by the HADS and the NLR.

**Electronic supplementary material:**

The online version of this article (doi:10.1186/s12871-015-0167-9) contains supplementary material, which is available to authorized users.

## Background

Determining and understanding predictive factors of postoperative analgesic consumption may help to anticipate patient’s needs and prescribe well-targeted analgesia. Several authors studied predictive factors of the severity of postoperative pain and highlighted the following elements: younger age, female gender, type of surgery, incision length, quantitative sensory testing, severity of preoperative pain, use of analgesics before surgery, psychological background, and genetic characteristics [1–3]. In that endeavour, little attention has been paid on the influence of two aspects related to postoperative analgesics requirements, i.e. the individual’s attitude towards painful situations and the status of his immune system. In this study, we implemented a new questionnaire, the Situational Pain Scale (SPS), to measure an individual’s attitude towards imaginary potential painful situations. This questionnaire was calibrated with the one-parameter logistic Rasch model [4] and designed to be invariant for age and gender. We hypothesized that patient’s postoperative analgesic requirements may be related to his attitude towards painful situations and that the status of his immune system plays an important role in mediating this relationship.

To assess the status of the patient’s immune system, we used the neutrophil-to-lymphocyte ratio (NLR) that has been proposed as one of the most sensitive markers to stratify patients in terms of inflammation [5–9].

The chief aim of this study was to evaluate the predictive value of these two variables, in isolation and in combination with other variables, for the analgesic requirements during 48 h following laparoscopic cholecystectomy.

## Methods

### Study population

After IRB approval (Ethical Committee, n°2003/23DEC/219) (CEBH of the *Université catholique de Louvain*, Brussels, Belgium) and written informed consent, we followed a cohort of 60 patients who underwent a laparoscopic cholecystectomy between February 2011 and February 2012. The exclusion criteria were emergent cholecystectomy, change of surgical technique (laparotomy), inability to fill in the questionnaires (e.g., cognitive troubles, inability to understand French or English), malnutrition or morbid obesity (as defined as a body mass index of less than 18 or more than 30 kg/m^2^).

Based on preliminary observations, we expected to observe a difference of 1 ± 1 (SD) units on the SPS scale between groups with low or high analgesic requirements. Using the software G*Power 3.1 (downloaded from the Website www.psycho.uni-duesseldorf.de/abteilungen/aap/gpower3/) with the following input parameters (one tailed t–test, alpha error probability = 0.05, power = 0.90 and equal allocation ratio), we computed a required total sample size of 46 patients.

### Procedures

Pre-, intra- and postoperative care was standardized. Medical data, pain assessment, SPS and HADS questionnaires were collected. Anaesthetic protocol was standardized as postoperative analgesic protocol including IV morphine titration followed by paracetamol (up to 4 g/day) and tramadol 50 mg (up to 300 mg/day). All these procedures are detailed in Additional file 1.

### Patient assessments

#### Pre- and postoperative autoevaluation of pain

Autoevaluation is described as the most valid evaluation of pain, because of its subjective characteristics [10]. As proposed by these authors, we used a verbal rating scale (VRS), a simple and sensitive evaluation scale of five levels (no pain, mild, moderate, intense and excruciating pain), at rest and during movement (walking) [10].

#### Primary endpoint: analgesic consumption

The consumption of analgesics is usually expressed by morphine equivalence; we have chosen not to use this method because of the difficulty to find a reliable equianalgesic chart. Indeed, most of meta-analyses put in doubt the validity of the equianalgesic charts [11–13]. As described by Knotkova et al., the problems in these charts were more the methods of equivalence calculation and the interfering elements than the consideration of each analgesic dose by itself as a valuable endpoint [12]. Consequently, in this study, we quantified the analgesic consumption by counting the number of times the patient requested an analgesic. Each time the patient requested an analgesic corresponds to a “unitary dosage (UD)”. The total UD during the late postoperative period lasting 48 h represents his analgesic requirements.

#### The SPS: measuring patient’s attitude toward imaginary painful situations

By attitude, we understand a personal disposition, possessed to different degrees, which impels the individual to react to objects, situations, or propositions in ways that can be called favorable or unfavorable. Although attitudes are subject to change, their directions and strengths are sufficiently enduring over periods of time to justify treating them as personality traits [14].

The SPS was developed and validated simultaneously in a population of 100 healthy adults and 111 chronic pain patients. Here we shall briefly report the procedures and metric properties of this scale as they are fully described in Decruynaere’s PhD Thesis accessible on the Internet [15] at <http://dial.academielouvain.be/handle/boreal:5246>.

**Fig. 1:**
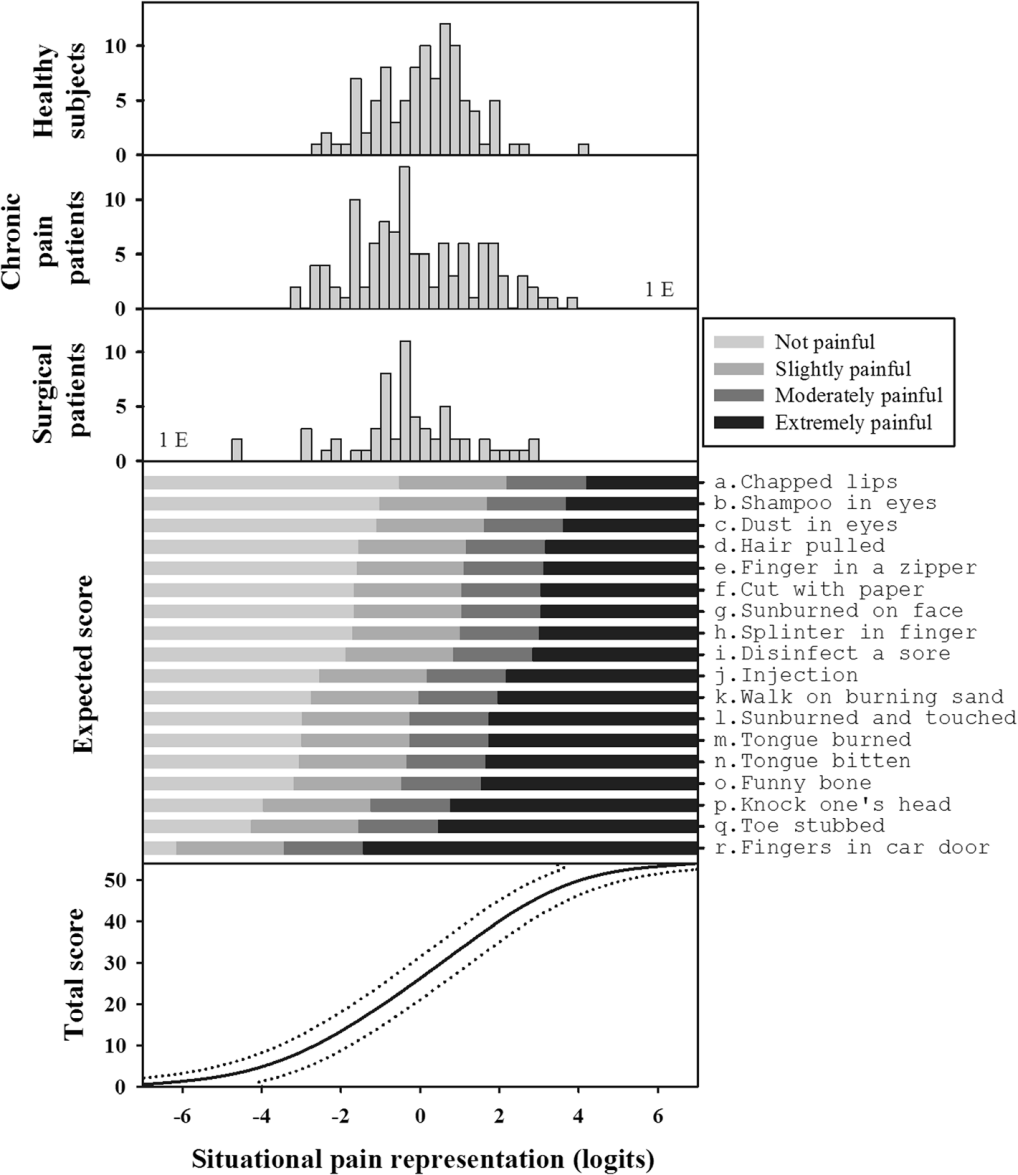
Three top panels: Distribution of the SPS measures of healthy subjects, chronic pain patients and surgical patients, respectively. Higher values are associated with higher pain intensity reports. Fourth panel: The item map providing a subject’s expected score to each item as a function of the measure of his pain attitude. Bottom panel: The relationship between raw scores and the pain attitude measures (solid line) and the 95 % confidence interval (dotted lines). The measures of pain attitude are obtained by converting the ordinal total scores on the 18 items into linear measures. For more details, see the main text and the Additional file 2

The SPS includes 18 items depicting imaginary painful situations administered as a self-reported questionnaire. These items are presented in Fig. 1 in order of situation painfulness. For each item, subjects were asked to estimate the pain intensity on a 4-level rating scale: not painful (0), slightly painful (1), moderately painful (2) or extremely painful (3). Moreover, subjects had the opportunity to rate situations as “impossible to estimate” whatever the reason (e.g., “never experienced”). This response was encoded as missing data. The 18 items contribute to the measure of a unidimensional variable and are invariant according to demographic (age and gender) and clinical subgroups (healthy and chronic pain patients) (Fig. 1, Appendix: Table 3). Methods, including references, for obtaining the SPS-score are detailed in the Additional file 2. Briefly, the Rasch rating scale model was used to calibrate the scale (item calibration). The expected responses to the items as functions of the measure of pain representation were compared to the painfulness of each item. We determined the most probable response of the subject to each item and compared it to the measures, expressed in logits. This final expression of the SPS score is available via the rehab-scale.org Internet site (http://www.rehab-scales.org/situational-pain-scale.html).

#### Anxiety and depression assessment

The preoperative state of anxiety and depression was evaluated one day before surgery by the Hospital Anxiety and Depression scale (HADS). This scale is a questionnaire composed by 7 items related to anxiety and 7 items related to depression [16]. A score over 10 for each dimension is considered as pathological. The French version has been validated [17].

#### NLR and inflammatory status assessment

The NLR has been proposed by cardiologists and in the perioperative period of cancer surgery as one of the most sensitive/specific biological markers to stratify patients in terms of inflammation [5, 6, 9]. The NLR is positively related to the inflammatory status and its consequences [7]. In our hospital, leukocytes count is typically included in the routine preoperative evaluation and prospectively registered in a computerized database. In this study, blood samples dated from 1 month or less before surgery, in stable conditions (e.g. after the resolution of any septic disorder). All venous blood samples were processed in a blood analyzer (Sysmex [TOA Medical Electronics, Kobe, Japan]) for the determination of the complete blood cell counts and differential counts of leukocytes. We recorded the neutrophils and the lymphocytes absolute counts, and calculated the NLR.

### Statistical analysis

The variables included in the multivariable regression and statistical analyses were either the ones that were found to distinguish the group of low consumers from high consumers or those that showed a strong association with worst pain intensity (Table 1). Additionally, univariate regression analyses assisted in the final selection for multiple regression models (Table 1). To control for the influence of multicollinearity, we computed the variance inflation factor (VIF) for every independent variable. The average VIF was 1.301 [range 1.190–1.440] well below the threshold for causing problems in one’s analysis [18, 19].

**Table 1:** Basic description of patient population and univariate analysis of potential predictors for postoperative analgesic requirements (mean ± SD)

Variables	All patients	Low consumers	High consumers	Difference	F	*P*
Analgesic requirements (UD)	3.5 ± 2.35	0 – 3	≥ 4			
*n*	60	31	29			
Gender Male/Female	21/39	13/17	8/22		1.64 ^a^	0.157
Age (years)	57.4 ± 17.9	60.7 ± 15.8	54.1 ± 19,4	6.67	2.131	0.150
SPS (Logits)	−0.30 ± 1.55	−0.83 ± 1.87	0.15 ± 1.44	−0.98	5.140	0.027
HADS total	14.1 ± 6.42	12.4 ± 5.21	16.1 ± 6.9	−3.67	5.345	0.024
HADS anxiety	4.9 ± 3.86	8.13 ± 4.06	10.2 ± 4.24	−2.07	3.722	0.059
HADS depression	9.1 ± 4.24	3.97 ± 3.21	5.9 ± 4.20	−1.93	4.006	0.050
VRS preop. at rest	0.70 ± 0.93	0.53 ± 0.90	0.87 ± 0.94	−0.33	1.975	0.165
VRS preop. during movement	0.88 ± 1.08	0.63 ± 1.07	1.13 ± 1.04	−0.50	3.376	0.071
Neutrophils (counts/mm^3^)	61.1 ± 11.27	64.6 ± 12.5	58.3 ± 9.9	6.29	4.734	0.034
Lymphocytes (counts/mm^3^)	27.2 ± 10.0	23.6 ± 9.91	30.9 ± 8.5	−7.3	9.380	0.003
Neutrophil/Lymphocyte Ratio	2.97 ± 2.52	3.81 ± 3.24	2.13 ± 0.98	1.68	7.376	0.009
Ln(NLR)	0.87 ± 0.61	1.09 ± 0.67	0.66 ± 0.46	0.43	8.606	0.005
Surgery duration (min)	79.0 ± 31.1	76.4 ± 31.9	82.6 ± 30.6	−6.17	0.585	0.447
IV Morphine (mg)	4.7 ± 5.07	4.5 ± 4.9	4.91 ± 5.3	−0.41	0.095	>0.500
Patients receiving opioids in the PACU (n)	39	19	20			>0.500

^a^Chi-Square statistic of independence for a 2x2 contingency table. UD: Unitary dosage. SPS: Situational pain scale. HADS: Hospital anxiety and depression scale. VRS: Verbal rating scale. NLR: Neutrophil/Lymphocyte Ratio. IV: Intravenous. PACU: Post-anesthetic care unit

**Fig. 2:**
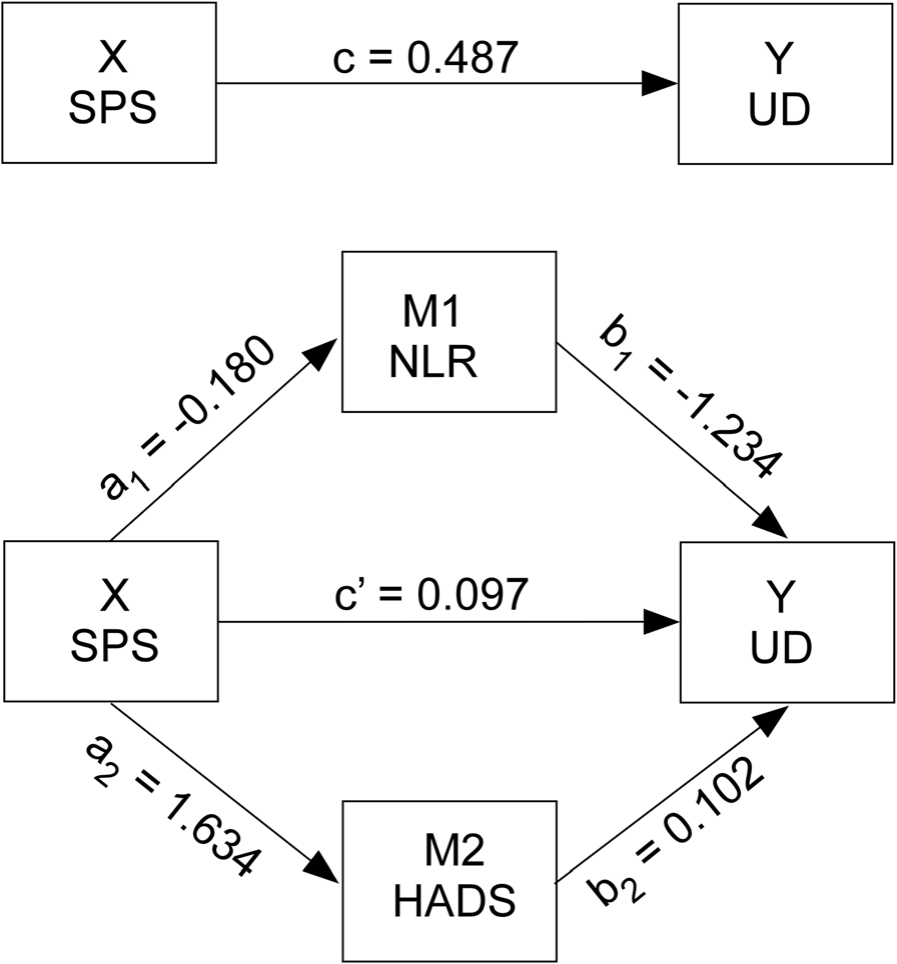
Upper panel: The path diagram represents the regression model whit c as the direct effect of SPS on UD. Lower panel: The path diagram represents the two mediator model where a_1_ and b_1_ are the coefficients of the indirect effect across NLR, a_2_ and b_2_ the coefficients of the indirect effect across HADS and c’ the residual effect of SPS on UD without mediation

For mediation analysis, a distinction between the various direct and indirect effects and their corresponding weights was performed (Fig. 2). To circumvent recognized issues with methods for testing mediation, Preacher and Hayes bootstrapping method was used [20]. The total effect of SPS scores on postsurgical analgesic consumption (UD) (weight c) consists of both a direct effect of pre-surgical attitude on postsurgical analgesic consumption (weight c’), and also indirect effects through mediators like NLR (weight a_1_xb_1_) and HADS (weight a_2_xb_2_). The effect of SPS on NLR is represented by weight a_1_, whereas weight b_1_ is the effect of NLR on postsurgical analgesic consumption. Similarly, the effect of SPS on HADS is represented by weight a_2_, whereas weight b_2_ is the effect of HADS on postsurgical analgesic consumption. Point estimates and 95 % bias-corrected and accelerated bootstrapped standard errors were estimated with 5000 bootstrap resamples. Statistical mediation analysis was performed with M*plus *[21] (V 6.12).

## Results

All the 60 patients completed the study and no missing values were in the collected data.

### Population characteristics

The population characteristics (age, gender, SPS, NLR, HADS, VRS, surgery duration) as analgesics requirements are reported in Table 1. Although the group of males was on average older (62.8 years) than the females (54.7 years) the mean difference of 8.2 years did not reach statistical significance (F = 2.884; P = 0.095). The mean (±SD) scores for anxiety and depression on the HADS test were 4.9 ± 3.9 and 9.1 ± 4.2, respectively. There was no statistical difference in mood between genders (F = 0.052; P > 0.5). Thirty-three (55 %) patients didn’t receive any opioids during surgery, but some of these patients received opioids in the recovery room so that finally only 8 patients were not exposed to opioids before returning to their room (Table 1).

### Pre-surgical patients and the situational pain scale

The distribution of measures for the attitude towards painful imaginary situations (SPS) for the pre-surgical patients is shown in Fig. 1 (third panel). These measures range from approximately −4.5 to 3 Logits, with higher values associated with higher pain intensity reports.

### Univariate analyses of potential predictors for postoperative analgesic consumption

The univariate analysis (Table 1) revealed a significant association between postoperative analgesic consumption (UD) and the following variables: SPS, total HADS scores, preoperative neutrophil counts, lymphocyte counts and the NLR. A negative relationship between SPS and the NLR (Ln(NLR) = 0.820–0.180*SPS; R^2^ = 0.211; F = 15.52; *P* < 0.001) and a positive relationship between SPS and the total score on the HADS (HADS = 14.8 + 1.63*SPS; R^2^ = 0.159; F = 10.97; *P* = 0.002) was observed. The logarithmic transformation of NLR was used as it markedly reduced the positively skewed distribution.

No significant associations were observed between UD and gender, age, preoperative pain intensity at rest and during movement measured by the VRS at rest and during movements, surgery duration and opioid administration (intraoperative period and in the Post Anaesthesia Care Unit).

### Multivariate analysis of analgesic consumption (primary endpoint) and maximum pain on the VRS (secondary endpoint) in the postoperative period

A multiple linear regression analysis based on the variables identified as significantly related to postoperative analgesic consumption yielded the following results:

UD = 3.18 + 0.10 * SPS − 1.24 * NLR + 0.10 * HADS; R^2^adj = 0.253; F_3,56_ = 6.309; *P* < 0.001. The contribution of NLR to the determination of UD was clearly the most effective.

The same analysis performed with the maximum pain on the VRS during the same postoperative period showed that this relation was clearly weaker as only 9.5 % of the variance in pain intensity was explained by the independent variables:

$$ \mathrm{VRSmax} = 1.74 + 0.08*\mathrm{S}\mathrm{P}\mathrm{S}\hbox{--} 0.24*\mathrm{N}\mathrm{L}\mathrm{R} + 0.02*\mathrm{HADS};\ {\mathrm{R}}^2\mathrm{a}\mathrm{d}\mathrm{j} = 0.095;\ {\mathrm{F}}_{3,56} = 3.074;P = 0.035 $$

In the following section, we present a statistical mediation analysis for examining more precisely the relationship among the predictor variables of postoperative analgesic consumption.

### Statistical mediation analysis

We followed Baron and Kenny’s steps [for a comprehensive review see [22]] for examining mediation in the present set of variables with the two-mediator model illustrated in Fig. 2. First step, the independent variable X (SPS) must affect the dependent variable Y (UD), i.e. the correlation coefficient c in the upper part of Fig. 2. That relationship was indeed significant (R = 0.321: *P* = 0.012). Second step, the independent variable X (SPS) must affect the first mediator (M_1_ or NLR) coefficient a_1_ and must affect the second mediator M_2_ or HADS) coefficient a_2_. These effects were both highly significant (see Table 2) implying that both variables were mediators of the relation between SPS and UD. Third, the mediator must affect the dependent variable (UD) when the independent variable (SPS) is controlled: coefficient b_1_ for the first mediator (NLR) and b_2_ for the second mediator (HADS). For both mediators the coefficients are significant with *P* = 0.017 and *P* = 0.033, respectively. Fourth and finally, the direct effect c’ (lower part of Fig. 2) must be no significant. Consequently, there was clear evidence for a complete mediation since the direct effect was no significant (*P* > 0.5) but a_1_*b_1_ was significant (*P* = 0.005) although a_2_*b_2_ (*P* = 0.161) was not.

**Table 2:** Paths coefficients and statistics of the two-mediator model (see Fig. 2 lower panel)

Paths	SPS - > NLR	SPS - > HADS	NLR - > DU	HADS - > DU	SPS - > UD	SPS - > NLR - > UD	SPS- > HADS - > UD	SPS - > UD sum of effects	HADS < − > NLR Interaction among mediators	
Symbols	a_1_	a_2_	b_1_	b_2_	c'	a_1_ x b_1_	a_2_ x b_2_	(a_1_ x b_1)_+ (a_2_ x b_2)_	m2 < − > m1	Contrast^a^
Coefficient	−0.180	1.634	−1.243	0.102	0.097	0.224	0.166	0.390	−0.093	0.057
S.E.	0.038	0.247	0.365	0.063	0.361	0.079	0.119	0.196	0.435	0.047
t statistic	−4.774	6.612	−3.403	1.608	0.269	2.819	1.401	1.985	−0.214	1.219
*P* value	< 0.001	< 0.001	0.001	0.108	0.788	0.005	0.161	0.047	0.831	0.223

^a^Contrast hypothesis that the two indirect effects are equal

Complete results of the analysis are reported in Table 2. As already mentioned here above, the need for analgesics (UD) was significantly related to the attitude towards imaginary painful situations (SPS) (c = 0.49; R^2^ = 0.103; F = 6.68; *P* = 0.012). In other words, a 1 unit increase in the SPS was associated with about half a unit increase in UD. This total effect can be explained by the mediated effects through the general state of inflammation measured by the NLR and mood measured with the HADS. There was a statistically significant effect of SPS score on NLR (a_1_ = −0.180; R^2^ = 0.211; F = 15.52; *P* < 0.001) and on the HADS (a_2_ = 1.634; R^2^ = 0.159; F = 10.97; *P* = 0.002). SPS was associated with a reduction of −0,18 in the NLR mediator and 1.63 change in the HADS mediator. The effect of the NLR mediator (b_1_ = −1.243; F = 6.09; *P* = 0.017) and the HADS mediator (b_2_ = 0.102; F = 4.76; *P* = 0.033) on UD was statistically significant when controlling for SPS. A 1 unit change in the NLR mediator was associated with a −1.24 decrease in UD and a 1 unit increase in HADS was associated with a 0.10 increase in UD. The adjusted effect of SPS on UD was not statistically significant (c’ = 0.097; F = 0.21; *P* > 0.5) consistent with a random association of SPS and UD during the 48 h following surgery. Apparently the overall significant relation between SPS and UD was due too the effects of SPS on the mediators. There was a drop in the value of c’ (=0.097) compared with c (=0.487) of 0.390.

The estimates of the two mediated effects were equal to a_1_xb_1_ = 0.224 for mediation through NLR and a_2_b_2_ = 0.166 for the mediation through HADS. The total mediated effect of a_1_b_1_ plus a_2_xb_2_ = 0.390, which is equal to c–c’ = 0.487-0.097, so that a 1 unit increase in attitude was associated with a 0.39 effect on UD through the two mediating variables. In other words, the variance in UD explained by the score on the SPS was indirect and amounts to 46 % through mediator NLR and to 34 % through mediator HADS. The total mediated effect explained 80 % of the variance in UD.

Finally, there was no significant interaction between the two mediators (t = 1.219; *P* = 0.223).

## Discussion

The present study shows that preoperative pain-related attitudes, as assessed by the SPS, were associated with the postoperative analgesic requirements (UD). Moreover, a higher score of anxiety and depression (HADS), or a lower preoperative NLR (and its components: a low neutrophil or a high lymphocyte counts) were also associated with a higher UD. Finally, and importantly, the HADS and the NLR mediate conjointly the effect of the SPS on the UD. In other words, patients with the strongest scores on the SPS, i.e. those who anticipate the highest pain scores, showed the lowest inflammatory status (assessed by the NLR) and the worst mood status (assessed by the HADS) that mediate, at least in this series, the effect on postoperative analgesic consumption (UD).

Others studied predictive factors of severe postoperative pain. For example, Kalkman et al.[1] developed a prediction tool for the risk of early severe postoperative pain. They found that young age, female gender, outpatient, high preoperative pain score, anxiety and need for information, type of surgery and large incision size, all are predictive of severe postoperative pain. In their work, laparoscopic cholecystectomy was classified as a surgical procedure with “highest expected pain” [1]. More recently[23], preoperative Quantitative Sensory Testing (QST) has been shown to be better correlated with postoperative pain, than demographic and psychological factors like vulnerability, anxiety, depression, catastrophizing. Indeed, responses to experimental (thermal or electrical) pain stimuli explain up to 54 % of the variance of clinical postoperative pain. Adding the psychological variables to the multivariate regression analysis didn’t significantly increase the predictive power of the model. This led to hypothesize that there is multicollinearity between psychological and sensory variables. In the present study, we confirm and quantify multicollinearity between psychological variables and inflammatory response components. Nevertheless, our primary endpoint was here original, the analgesic needs (UD), in comparison with pain scores and/or the risk of severe postoperative pain, as described in the previous studies. Our analysis concerning the pain scores shows that, if the same relations cannot be excluded, these are clearly weaker than with the UD. Therefore, we identified here a potentially interesting new behavioural variable, associated with postoperative pain, but more sensitive in the context of the analysis of multicollinearity between psychological variables, inflammatory response and pain behaviour.

This multicollinearity comes not as a surprise as pain-related behaviour was linked to lymphocytes and neutrophil counts in psychological intervention on depressive symptoms in cancer patients. Indeed, using a mediation analysis, as in the present study, Thornton et al. [24] showed that the effect of psychological intervention on white blood cell count in breast cancer patients was mediated by the reduction of depressive symptoms. This effect was mostly apparent on a reduction of the neutrophils count, but also present on lymphocytes count. They hypothesized that psychological intervention was associated with a shift from a constantly over activate immune response to a more, and better responding, adaptive immune status. In that case, immune response should be characterized by lower lymphocytes and neutrophils counts in the basal state but an increased inflammatory response resolving rapidly during the postoperative phase.

Using preoperative relaxation technique, Manyande et al. [25] reported that a reduction of the anxiety before and after surgery induced a more robust cortisol and epinephrine response (typically associated with a stronger acute physiological stress response). Interestingly, in their study, postoperative pain scores were similar between the groups (relaxation or not) whereas the analgesic requirements were twice less in patients with relaxation intervention, but with stronger inflammatory response. We observed similarly an association between psychological variables, inflammatory response and postoperative analgesics requirements.

The increased preoperative inflammatory status we measured is concordant with the association seen between a high redistribution profile after an inflammatory event (i.e. high neutrophils counts and rapidly decreasing lymphocytes count after surgery) and a better recovery and functional status [26]. We can hypothesise that the patients with a preoperative low SPS score could have a more responsive adaptive immune response profile but this remains speculative and should be tested in further studies.

The exploratory way used is the main limitation of this work. The use of the SPS remains a new tool to assess attitude towards painful situations, as the endpoint proposed to assess the analgesics requirements (total UD during the postoperative period)(For details over procedure and SPS calibration, see Appendix: Table 3, Additional files 1 and 2). Nevertheless, the correlation between the SPS and the UD permits to describe logical associations in the absence of satisfactory alternative tools (for these types of status and behaviour assessments). Additionnally, regarding the difference between UD and analgesic requirements, one can argue that analgesics requirements are multifactorial and the number of analgesic requirements depends primarily on the type, the pharmacokinetics and pharmacodynamics of the analgesic(s) used. This is not considered primarly in this work, defining "patients' requests" for analgesia as primary endpoint. We consider as strengths the use of the NLR as a sensitive marker of the inflammatory status, the wide use of the HADS for the assessment of the emotional status, as the clear results obtained by the mediation analysis. Finally, we recognize that the adjusted comparisons on patient’s weight, while malnutrition and morbid obesity were exclusion criteria, would merit additional comparisons.

## Conclusions

We have shown, in this series of patients undergoing laparoscopic cholecystectomy, that the preoperative inflammatory status (assessed by the NLR) and the preoperative emotional status (assessed by the HADS) mediate conjointly the predictive value of attitude towards painful situations (assessed by the SPS) in postoperative analgesic requirements. These results emphasize the place of immune markers and related inflammatory scores, like the NLR, in perioperative pain studies.

## Appendix

**Table 3:** The Situational Pain Scale calibration

*The SPS Calibration*					
Item		Measure	SE	Mean square fit statistics	
		(Logits)	(Logits)	infit	outfit
a.	My lips are chapped	1.96	0.12	1.12	1.06
b.	I get shampoo in my eye	1.45	0.12	1.00	0.95
c.	I get a speck of dust in the eye	1.38	0.12	1.20	1.19
d.	Someone pulls my hair	0.92	0.12	1.00	0.98
e.	I catch my finger in a zipper	0.87	0.12	1.06	1.03
f.	I cut myself with a sheet of paper	0.81	0.12	1.03	1.01
g.	I get sunburned on my face	0.81	0.11	1.03	1.02
h.	I have a splinter under the skin of one finger	0.77	0.11	1.12	1.14
i.	I disinfect a sore	0.60	0.11	1.10	1.12
j.	I get an injection in the arm	−0.60	0.11	1.12	1.11
k.	I walk on burning sand	−0.27	0.11	1.16	1.17
l.	I get sunburned and someone touches me on that spot	−0.50	0.11	0.96	0.99
m.	I burn my tongue tasting scorching hot food	−0.51	0.11	0.76	0.77
n.	I bite my tongue	−0.58	0.11	0.77	0.79
o.	I hit my funny bone	−0.71	0.11	1.04	1.03
p.	I knock my head on the corner of a piece of furniture	−1.48	0.11	0.75	0.77
q.	I stub my toe on a chair leg	−1.78	0.11	0.91	0.93
r.	I get my fingers caught in the car door	−3.67	0.16	0.86	0.90
Mean		0.00	0.12	1.00	1.00
S.D.		1.33	0.01	0.14	0.13

## Additional files

Additional file 1:
**Detailed intra-, pre- and postoperative procedures.** (DOC 25 kb) Additional file 2:
**Calculation of the SPS-score.** (DOC 29 kb)
