# Supplementary material for: Biological inflammatory markers mediate the effect of preoperative pain-related behaviours on postoperative analgesics requirements
Source: BMC Anesthesiol. 2015 Dec 16;15:183. doi: 10.1186/s12871-015-0167-9 (PMC4681155; doi:10.1186/s12871-015-0167-9)
Supplement: Additional file 1: — Detailed intra-, pre- and postoperative procedures. (DOC 25 kb) [file 12871_2015_167_MOESM1_ESM.doc]

**Additional file 1.** Detailed intra-, pre- and postoperative procedures

There were four periods in the patient’s care: the preoperative, the intraoperative, the early and the late postoperative period. The preoperative period consisted of the day before surgery, when a short anamnesis was done, as well as recording of the usual medication and pain assessment at rest and at movement using a 5 level verbal rating scale (VRS)^1^. The patient filled in the SPS and the HADS questionnaires. The junior anaesthetist informed the patient about the management of postoperative pain, explaining that analgesics shall be given on demand. During the intraoperative period, a standard anaesthetic protocol was used, if necessary adjusted according to patient’s needs. Typically, patients received a target controlled continuous infusion of propofol, for a bispectral index between 40 and 60 - induction of general anaesthesia by slow intravenous route: clonidine 300 µg, ketamine 0.5mg/kg, MgSO4 3g, ketorolac 30mg and morphine 0.05mg/kg at the end of the surgical procedure. Three surgical techniques are possible: classic laparoscopy (4 incisions, instruments standard size), microinstrumental laparoscopy (4 incisions, microinstruments), and single port access laparoscopy (single umbilical incision). Laparoscopic cholecystectomy could be converted at any moment into a laparotomy, even if it was not the case in any of these patients. Information concerning the indication of surgery, the type of surgical technique, the drain placement, the protocol of anaesthesia, the duration of anaesthesia and the duration of surgery were recorded.

The early postoperative period consisted of approximately 2 hours surveillance in the Post Anaesthesia Care Unit (PACU). Analgesia was managed by nurses, under medical supervision, and consisted of titration of morphine in the PACU. Finally, the late postoperative period began when the patient returned to his room and extended over the following two days. Well-trained nurses evaluated the pain with a 5 levels VRS. This evaluation occurred on every nursing tour, on average every three hours. The nurses administered analgesics on the patient’s demand (paracetamol up to 4 g/day and tramadol 50 mg up to 300 mg/day). Each administrated analgesic was compulsorily recorded in the patient’s file so as to provide the exact quantity of analgesics consumed during that late postoperative period.
