# Supplementary material for: Biological inflammatory markers mediate the effect of preoperative pain-related behaviours on postoperative analgesics requirements
Source: BMC Anesthesiol. 2015 Dec 16;15:183. doi: 10.1186/s12871-015-0167-9 (PMC4681155; doi:10.1186/s12871-015-0167-9)
Supplement: Additional file 2: — Calculation of the SPS-score. (DOC 29 kb) [file 12871_2015_167_MOESM2_ESM.doc]

**Additional file 2. Calculation of the SPS-score**

The Rasch rating scale model ^2 3^ used to calibrate the scale provides a measure of subject’s attitude toward painful imaginary situations and the painfulness of each situation (item calibration) ^4 5^. Distribution of measures of the healthy and the chronic pain population used to calibrate the scale is shown on the two top panels of figure 1. Location of items and thresholds that separate each pair of adjacent response categories is shown on the fourth panel of figure 1. This panel displays the expected response to a given item as a function of the measure of pain representation. By comparing the measure of a given subject to the painfulness of each item, it is possible to determine the most probable response of the subject to each item. All these measures can be located on the same measurement scale and expressed in the same units. This unit is the logit, the natural logarithm of the odds for perceiving a particular situation as painful. The odds represent the ratio of the probability of rating the situation in one category to the probability of rating the same situation in the category just below (e.g., moderately painful vs. slightly painful). Consequently, subjects who report high pain intensity ratings have measures of greater magnitude and are located on the right of the scale. The rating scale model estimates the measures from the raw responses of the subjects to the items. The relationship between raw scores and attitude measures of subjects is depicted by an ogival relationship (bottom panel of figure 1). The transformation of raw scores into the Rasch pain attitude measures or SPS scores can be obtained via the rehab-scale.org Internet site (<http://www.rehab-scales.org/situational-pain-scale.html>).
